# Neural correlates of intentional and stimulus-driven inhibition: a comparison

**DOI:** 10.3389/fnhum.2014.00027

**Published:** 2014-02-05

**Authors:** Margot A. Schel, Simone Kühn, Marcel Brass, Patrick Haggard, K. Richard Ridderinkhof, Eveline A. Crone

**Affiliations:** ^1^Institute of Psychology, Leiden UniversityLeiden, Netherlands; ^2^Leiden Institute for Brain and CognitionLeiden, Netherlands; ^3^Max Planck Institute for Human DevelopmentBerlin, Germany; ^4^Department of Experimental Psychology, Ghent UniversityGhent, Belgium; ^5^Institute of Cognitive Neuroscience, University College LondonLondon, UK; ^6^Department of Psychology, University of AmsterdamAmsterdam, Netherlands; ^7^Cognitive Science Center AmsterdamAmsterdam, Netherlands

**Keywords:** response inhibition, action, volition, fMRI, cognitive control

## Abstract

People can inhibit an action because of an instruction by an external stimulus, or because of their own internal decision. The similarities and differences between these two forms of inhibition are not well understood. Therefore, in the present study the neural correlates of intentional and stimulus-driven inhibition were tested in the same subjects. Participants performed two inhibition tasks while lying in the scanner: the marble task in which they had to choose for themselves between intentionally acting on, or inhibiting a prepotent response to measure intentional inhibition, and the classical stop signal task in which an external signal triggered the inhibition process. Results showed that intentional inhibition decision processes rely on a neural network that has been documented extensively for stimulus-driven inhibition, including bilateral parietal and lateral prefrontal cortex and pre-supplementary motor area. We also found activation in dorsal frontomedian cortex and left inferior frontal gyrus during intentional inhibition that depended on the history of previous choices. Together, these results indicate that intentional inhibition and stimulus-driven inhibition engage a common inhibition network, but intentional inhibition is also characterized by additional context-dependent neural activation in medial prefrontal cortex.

## Introduction

In daily life, most people experience and exercise a degree of voluntary control over their actions. The concept of intentional action is well recognized in the neuroscience literature. Several studies have focused on the voluntary choice between alternative actions (the so-called “what-component” of intentional action generation), and the voluntary choice of when to initiate action (the “when–component”) (Brass and Haggard, 2008). Neuroimaging research has shown that the processes of intentional action selection and planning are supported by a medial prefrontal network, including the rostral cingulate zone and pre-supplementary motor area (preSMA) (Lau et al., 2004, 2006).

A recent novel line of research has suggested that the inhibition of actions, like the generation of actions, can also be either intentionally driven or stimulus-driven. Intentional inhibition has been conceptualized as a late “veto-process,” a final check-and-brake function before action execution (Kühn et al., 2009; Filevich et al., 2012). It has been proposed as a third component in models of intentional action generation, the so-called “whether–component” (Brass and Haggard, 2008). In contrast to the “what” and “when” components of the model of intentional action generation, the “whether” component is difficult to examine, especially on a behavioral level, since intentional inhibition (i.e., internally driven inhibition) involves no external imperative stimulus, and does not result in any overt behavior. Two recent studies aimed to investigate intentional inhibition by asking participants to prepare actions, but then to occasionally cancel them at the last possible moment prior to action. These studies revealed a distinct neural network that was more activated in intentional inhibition than in intentional action, including the dorsal frontomedian cortex (dFMC) (Brass and Haggard, 2007; Kühn et al., 2009).

In contrast to the scarce literature on intentional inhibition, most studies of action inhibition have focused on stimulus-driven inhibition (i.e., externally driven inhibition). Within neuroscience research stimulus-driven inhibition has been extensively studied using different paradigms, such as go/nogo tasks (Casey et al., 1997) and stop-signal tasks (Logan and Cowan, 1984). In these paradigms an external stimulus signals that participants have to inhibit a prepotent or already prepared response. Successful performance on these stimulus-driven inhibition paradigms appears to rely on a fronto-striatal network (Aron and Poldrack, 2006; Verbruggen and Logan, 2008; Aron, 2011; Ridderinkhof et al., 2011). Within this network, specifically, the right inferior frontal gyrus (rIFG) and preSMA have been implicated as crucial for the inhibition of motoric responses (Aron et al., 2004; Chikazoe, 2010; Jahfari et al., 2011, 2012). Importantly, stimulus-driven inhibition is influenced by preceding contexts, such that participants are more likely to make errors in inhibiting when an inhibition trial is preceded by a larger number of go-trials (Durston et al., 2002a,b). Also activation in key regions, such as the rIFG becomes stronger during inhibition following a larger number of go-trials (Durston et al., 2002a,b). Stimulus-driven inhibition benefits from a number of distinct methodological advantages, including a well-circumscribed experimental task, and its mechanisms and dynamics are detailed by well-developed computational models (Aron and Poldrack, 2006). However, one recent review has noted that stimulus-driven inhibition may not capture the crucial operations of cognitive inhibitory control in everyday life, and particularly in social contexts. Explicit stop-signals are relatively rare in real life, and society (including legislation) assumes that healthy adults have the capacity to decide for themselves when to refrain from an action (Aron, 2011).

Despite the large literature on stimulus-driven inhibition, to date no study directly compared stimulus-driven inhibition and intentional inhibition. Nevertheless, understanding whether self-generated decisions to inhibit action are different from stimulus-driven decisions, remains an important question, both for the scientific understanding of inhibitory control, and for potential therapies for conditions such as impulsivity, harmful behavior, or shyness. In particular, is the neural network supporting stimulus-driven inhibition (lateral prefrontal cortex/preSMA) also involved in intentional inhibition, or is a different neural network involved in intentional inhibition (including dFMC)? Additionally, is intentional inhibition dependent on preceding context, as has been previously observed for stimulus-driven inhibition (Durston et al., 2002a,b)?

The present study is the first to test the neural correlates of intentional and stimulus-driven inhibition within the same subjects. To this end, participants performed two inhibition tasks while lying in the scanner; the marble task to measure intentional inhibition (Kühn et al., 2009) and the stop-signal task to measure stimulus-driven inhibition (Logan and Cowan, 1984). In the marble task, participants have to intentionally inhibit an externally triggered prepotent response. A marble begins to roll down a slope. If the marble turns green as it begins to roll, they must rapidly press a button to stop it from rolling down. If the marble remains white, they may choose whether to press and stop it, or inhibit pressing and let it roll down. The contrast of crucial interest for the marble task was the contrast between the two possible outcomes of intentional decisions: i.e., inhibition vs. action. We hypothesized that this contrast would show additional neural activity in dFMC as was previously shown by Kühn et al. (2009). Interestingly, this activation is not normally reported in the equivalent contrast for stimulus-driven inhibition. Secondly, in the current study, the marble task was used to identify the neural network supporting the intentional inhibition decision process, by contrasting trials in which participants intentionally decide to inhibit with trials in which participants are instructed to respond (green marble trials). These neural regions were compared with the contrast of successful stopping vs. executing an action in the stop signal task by means of a conjunction analysis. We hypothesized that the fronto-striatal inhibition network (Aron and Poldrack, 2006; Ridderinkhof et al., 2011) would be involved in both the intentional and the stimulus-driven inhibition decision process.

## Methods

### Participants

Twenty-four healthy right-handed adults between 18 and 26 years of age (13 females, *M* = 21.49, *SD* = 2.36) participated in the experiment. All participants had normal or corrected-to-normal vision, and no neurological or psychiatric impairments according to self-report. Before participating in the experiment, all participants signed informed consent. In accordance with guidelines of the Leiden University Medical Center, all anatomical scans were reviewed by a radiologist. No anomalous findings were reported. To obtain an estimate of cognitive functioning participants completed two subtests of the Wechsler Adult Intelligence Scale (WAIS) (Wechsler, 1981); similarities and block design. Estimated IQ scores were within the normal range (*M* = 111.33, *SD* = 6.93).

### Experimental tasks

Participants performed two response inhibition tasks while lying in the MRI scanner. The tasks were presented in a fixed order. Participants first performed the marble task as a measure of intentional inhibition, followed by the stop-signal task as a measure of stimulus-driven inhibition.

#### Marble task

**Figure 1:**
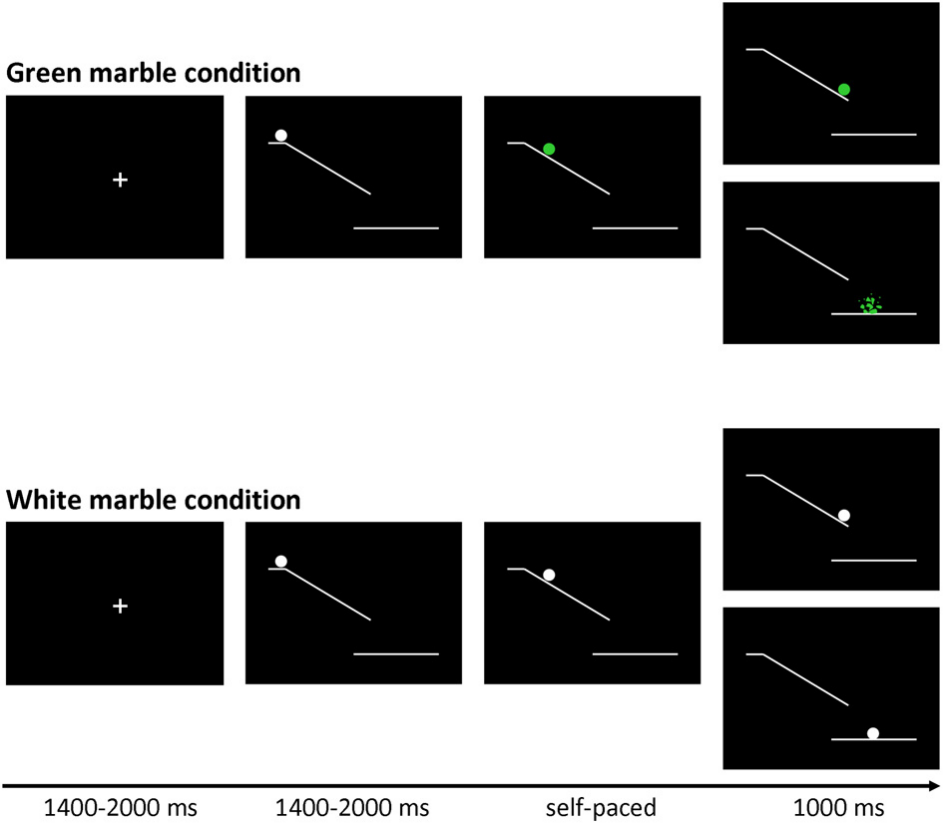
**Trial structure of the marble task.** Stimuli were presented on a black background. At the beginning of each trial a white marble on top of a ramp was presented. After a variable delay (jittered between 1400 and 2000 ms) the marble started to roll down the ramp, and could change color to green.

The marble task was adapted from Kühn et al. (2009). Each trial (see Figure 1) started with the presentation of a fixation screen (white cross against a black background) with duration jittered between 1400 and 2000 ms. The fixation screen was followed by a screen showing a white ramp with a white marble on top presented against black background. After a variable duration of 1400 to 2000 ms the marble started rolling down the ramp and participants could stop the marble from crashing by pressing a button. Finally, a feedback screen, showing trial outcome, was presented for 1000 ms. There were two task conditions: a green marble and a white marble condition.

In the green marble condition, the white marble changed to green as soon as it started rolling. The task was programmed in such a way that participants viewed 16 rapidly presented static pictures showing the marble at successive locations on the ramp, which was experienced as a rolling movement. Participants were instructed to stop the marble from crashing by pressing a response button with their right index finger. When participants were successful at stopping the marble, they were presented with a feedback screen showing the location where they had stopped the marble. When participants were not successful at stopping the marble, they were presented with a feedback screen showing a shattered marble beneath the ramp. The speed of the marble was adjusted by a staircase-tracking procedure. At the start of the experiment, the static pictures were presented for 30 ms each. When participants were successful at stopping the marble the duration was decreased with 10 ms, making the task more difficult. When participants were not successful at stopping the marble in time the duration was increased with 10 ms, making the task easier. The staircase procedure was allowed to fluctuate between 20 and 80 ms, allowing a response window between 320 and 1280 ms.

In the white marble condition, the marble did not change color and participants were instructed to choose between responding and inhibiting. When participants responded, they were presented with a feedback screen showing the location where they had stopped the marble. When participants inhibited, they were shown a feedback screen showing the white marble at the bottom of the ramp. In order to motivate participants to balance the frequency of responding and inhibiting, they were told that the stopped and non-stopped marbles would fall in different baskets. Participants were instructed to collect an equal amount of marbles in each basket, but were not allowed to count or use a sequencing strategy; therefore participants were instructed to make an independent decision every time the marble stayed white. At the end of each block participants were shown how many marbles they had collected in each basket. As will be described in the results section, the participants were successful in following the instruction to stop the marble on approximately 50% of the trials.

In order to give participants sufficient time to decide between responding and inhibiting the speed of the white marble rolling down the ramp was set considerably slower. The speed of the sequentially presented static white marble pictures was set to the speed currently reached in the green marble condition plus 30 ms. Consequently the duration of the sequentially presented static white marble pictures was allowed to fluctuate between 50 and 110 ms, allowing a response window between 800 and 1760 ms.

The experiment consisted of three blocks of 80 trials, each block consisting of 48 green and 32 white marble trials. Trials were presented in a pseudo-randomized order so that each white marble trial was preceded by 0, 1, 2, or 3 green marble trials. The large proportion of fast-paced green trials served two functions. First, the fast-paced green trials lead to a prepotent tendency for action. This was desirable, so that intentional inhibition of action would involve a late brake on an already-prepared action, rather than an early decision not to initiate action preparation. Second, the randomized interleaving of intentional (white) and instructed (green) trials discouraged participants from strategically pre-deciding a pattern of intentional action, such as act-inhibit-act-inhibit etc.

#### Stop-signal task

The stop-signal task (Logan and Cowan, 1984) was presented in a visual form. Each trial started with the presentation of a green left- or rightwards pointing arrow. Participants were instructed to make a speeded response to the direction of the arrow, for the leftwards pointing arrow participants had to press a button with their left index finger and for the rightwards pointing arrow participants had to press a button with their right index finger. The arrow disappeared when participants responded or after 1500 ms had passed. Following the presentation of the arrow a fixation cross was presented with a duration jittered between 2000 and 4000 ms. When participants responded to the arrow, the duration of the fixation cross was extended by 1500 ms minus the reaction time, in order to keep the duration of the task stable between participants.

On a limited number of stop-trials (25%) a stop-signal was presented. In this case the arrow suddenly changed color to red. This color change indicated that participants had to inhibit responding to the direction of the arrow. Stop-signal delay (SSD) was adjusted using a staircase-tracking procedure to guarantee a 50% inhibition rate (Lappin and Eriksen, 1966). At the beginning of the task SSD was set at 250 ms. When participants successfully inhibited SSD was increased by 50 ms to make the task more difficult, when participants were not able to inhibit responding SSD was decreased by 50 ms to make the task easier.

The experiment consisted of two blocks of 128 trials, each block consisting of 96 go-trials and 32 stop-trials. Trials were presented in a pseudo-randomized order so that each stop-trial was preceded by 1, 2, 4, or 5 go-trials.

### Data acquisition

Scanning was performed with a standard whole-head coil on a 3.0 Tesla Philips scanner at the Leiden University Medical Center. The marble task consisted of 3 event-related runs, each lasting approximately 6 min, and the stop-signal task consisted of 2 event-related runs, both lasting approximately 5 min. Functional data were acquired using T2^∗^-weighted echo-planar imaging (EPI). The first 2 volumes of each run were discarded in order to allow for equilibration of T1 saturation effects (*TR* = 2.2 s, *TE* = 30 ms, sequential acquisition, 38 slices of 2.75 mm, field of view 220 mm, 80 × 80 matrix, in-plane resolution 2.75 mm). After the functional runs a high-resolution 3D T1-FFE scan for anatomical reference was obtained (*TR* = 9.760 ms; *TE* = 4.59 ms, flip angle = 8°, 140 slices, 0.875 × 0.875 × 1.2 mm^3^ voxels, field of view =224 × 168 × 177 mm^3^). Head motion was restricted by using foam inserts between the head and the head coil. Visual stimuli were projected onto a screen in the magnet bore that could be viewed through a mirror attached to the head coil.

### Behavioral data analysis

For the marble task, repeated measures analyses of variance were performed to examine the effect of preceding context on intentional inhibition. Planned comparisons were performed between the different numbers of preceding green trials, to examine which conditions differed from each other.

The use of response selection strategies on the marble task was evaluated by computing the Random Number Generation 2 (RNG2) index using the program RgCalc (Towse and Neil, 1998). The RNG2 index is an adaptation of the RNG index (Evans, 1978) optimized for two-choice response sequences, which considers the randomness of the sequence (Neuringer, 1986). RNG2 scores can range from 0 (null predictability) to 1 (complete predictability).

For the stop-signal task, the Stop signal reaction time (SSRT) was calculated according to the horse-race model of stopping (Logan and Cowan, 1984) following the procedures described in Band et al. (2003). In short, first all reaction times (RTs) for the correct go-trials were rank-ordered. Next, the percentage of failed inhibition was determined. Then, the go-RT corresponding to that percentage was determined. Finally, SSRT was computed as the difference between the go-RT corresponding to the percentage of failed inhibition and the mean SSD.

### fMRI data analysis

Data were preprocessed using SPM8 (Wellcome Department of Cognitive Neurology, London). Images were corrected for rigid-body motion. Structural and functional volumes were spatially normalized to T1 templates. The normalization algorithm used a 12-parameter affine nonlinear transformation involving cosine basis functions, and then resampled the volumes to 3-mm cubic voxels. Translational movement parameters never exceeded 1 voxel (<3 mm) in any direction for any subject or scan. Templates were based on the MNI305 stereotaxic space (Cocosco et al., 1997), an approximation of Talairach space (Talairach and Tournoux, 1988). Functional volumes were spatially smoothed with an 8-mm full-width-at-half-maximum isotropic Gaussian kernel. Statistical analyses were performed on individual participants' data using the general linear model in SPM8. The fMRI time series data were modeled by a series of events convolved with a canonical hemodynamic response function (HRF) and the temporal derivatives. For the marble task, the onset of marble motion of each trial was modeled as an event of interest. Separate regressors were defined for white nogo (intentional inhibibition), white go (intentional action), green go (stimulus-driven action), and green omissions (omission on the green marble trials). For the stop signal task, the presentation of the arrow of each trial was modeled as an event of interest. Separate regressors were defined for stop-successful, stop-unsuccessful, go-successful, and go-unsuccessful trials. The trial functions were used as covariates in a general linear model, along with a basic set of cosine functions to high-pass filter (120 Hz) the data. The least-squares parameter estimates of the height of the best-fitting canonical HRF for the different conditions were used in pair-wise contrasts. All reported effects consisted of at least 10 contiguous voxels that exceeded a false-discovery-rate (FDR) corrected threshold of *p* < 0.05, unless otherwise specified.

To examine similarities across contrasts, conjunction analyses were computed using the minimum statistic approach (Nichols et al., 2005). These analyses identified clusters that were significantly engaged at our threshold in both contrasts that we examined.

Region of interest (ROI) analyses were performed to further characterize the involvement of brain regions in intentional inhibition. ROI analyses were performed with the MarsBaR toolbox in SPM8 (Brett et al., 2002) (http://marsbar.sourceforge.net).

## Results

### Behavior

#### Marble task

**Figure 2:**
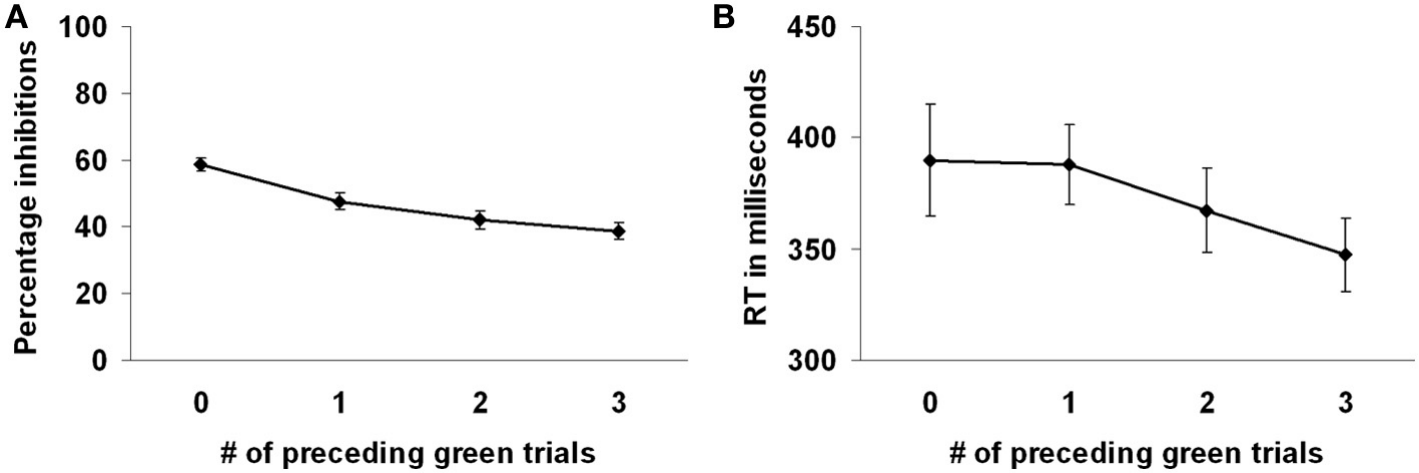
**Preceding context effects in the marble task. (A)** Participants inhibited more frequently with less preceding green trials. **(B)** Reaction times on the white marble trials were faster with more preceding green trials.

Participants successfully responded to the green marble on 63.22% of the trials. Participants intentionally inhibited responding to the white marble on 53.17% of the trials. Participants more often decided to inhibit responding to the white marble when there were fewer preceding green trials, *F*_(3, 69)_ = 18.09, *p* < 0.001 (see Figure 2A). That is, intentional inhibition decreased as the previous history of instructed go-responses increased. Planned comparisons showed that participants more often inhibited when there were 0 compared to 1, 2, or 3 preceding green trials (all *p*'s < 0.001) and when there was 1 compared to 3 preceding green trials (*p* < 0.05). The level of inhibition was comparable for the conditions where there were 1 or 2, and 2 or 3 preceding green trials (respectively, *p* = 0.14, *p* = 0.24).

To examine the randomness of response selection the RNG2 index was computed. A mean RNG2 index of 0.807 (*SD* = 0.012) was observed. To examine the randomness the participants' RNG2 index was compared with a RNG2 index computed over a set of randomly generated sequences of go- and nogo-responses. For the randomly generated set of go- and nogo-response sequences a mean RNG2 index of 0.801 (*SD* = 0.002) was observed. Although the RNG2 index for the randomly generated sequences was marginally but significantly smaller compared to the participants' RNG2 index, *F*_(1, 47)_ = 5.71, *p* < 0.05, these results indicate that the participants' behavior was close to being random and not driven by simple alternation strategies.

Reaction times were shorter for the green marble trials (*M* = 301, *SD* = 39) compared to the white marble trials (*M* = 372, *SD* = 89), *F*_(1, 23)_ = 32.55, *p* < 0.001, indicating that the decision process in the white marble trials took more time. However, longer reaction times on the white marble trials might also be partially related to the slower marble speed on those trials. Reaction times on the white marble trials were faster when there were more preceding green trials, *F*_(3, 69)_ = 5.52, *p* < 0.01 (see Figure 2B). Planned comparisons showed that reaction times were faster when there were 3 compared to 0 or 1 preceding green trials (respectively, *p* < 0.01, *p* < 0.001). Reaction times did not differ between the other conditions of preceding green trials (all *p*'s > 0.05).

#### Stop-signal task

Participants successfully responded to the direction of the arrow on 96.46% of the go-trials. The average reaction time on the successful go-trials was 519 ms (*SD* = 133). Participants successfully inhibited responding to the direction of the arrow on 46.03% of the stop-trials. SSRT was 281 ms (*SD* = 45).

#### Correlation between intentional and stimulus-driven inhibition

To examine the interrelations between the inhibition tasks, a correlation analysis was performed. Intentional inhibition as measured by the marble task (% intentional inhibition) was not correlated with stimulus-driven inhibition as measured by the stop-signal task (SSRT), *r* = 0.181, *p* = 0.40.

### fMRI results

#### Marble task

**Figure 3:**
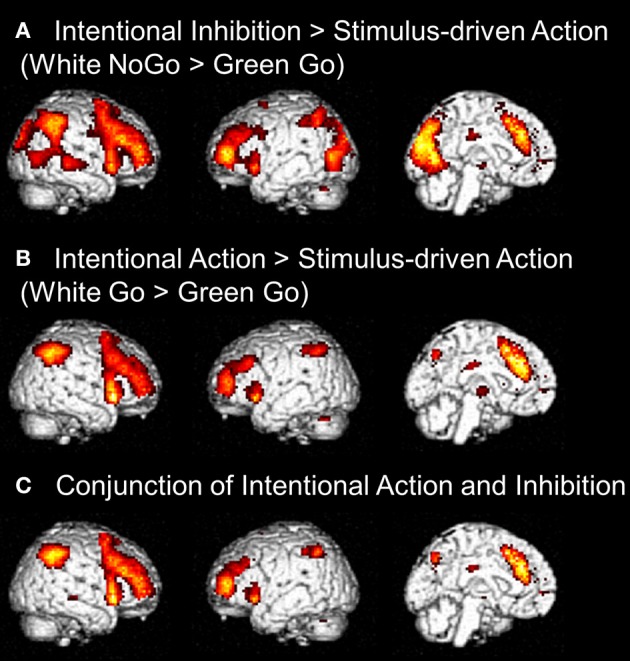
**(A)** Whole brain contrasts showing activation related to intentional inhibition decision process (White NoGo > Green Go) (FDR-corrected *p* < 0.05, at least 10 contiguous voxels). **(B)** Whole brain contrasts showing activation related to intentional action decision process (White Go > Green Go) (FDR-corrected *p* < 0.05, at least 10 contiguous voxels). **(C)** Conjunction analysis showing overlapping activation in intentional inhibition decision processes (White NoGo > Green Go) and intentional action decision processes (White Go > Green Go) (FDR-corrected *p* < 0.05, at least 10 contiguous voxels).

First, to identify the brain regions underlying the intentional inhibition decision process the contrast intentional inhibition > stimulus-driven action (White NoGo > Green Go) was computed. This analysis revealed activation in a widespread neural network (see Figure 3A and Table 1) consisting of bilateral IFG, bilateral middle frontal gyrus (MFG), bilateral superior frontal gyrus (SFG), preSMA/anterior cingulate cortex (ACC), bilateral inferior parietal lobule (IPL), right superior temporal gyrus (STG), and occipital lobe. Second, the brain regions underlying the intentional action decision process were identified by computing the contrast intentional action > stimulus-driven action (White Go > Green Go). This analysis revealed a similar activation pattern as the previous analysis, namely bilateral IFG, bilateral MFG, bilateral SFG, preSMA/ACC, and bilateral IPL (see Figure 3B and Table 1). To formally compare activation patterns related to the intentional inhibition decision process and the intentional action decision process a conjunction analysis was performed. This analysis confirmed the considerable overlap in brain regions underlying both intentional decision processes by revealing significant overlapping activation in bilateral IFG, bilateral MFG, bilateral SFG, preSMA/ACC, and bilateral IPL (see Figure 3C and Table 1).

**Table 1:** **Brain regions revealed by whole brain contrast, focused on decision processes (all FDR corrected, *p* < 0.05, >10 voxels)**.

**Anatomical region**	**L/R**	***K***	***Z***	**MNI coordinates**		
				***x***	***y***	***z***
**INTENTIONAL INHIBITION > STIMULUS-DRIVEN ACTION (WHITE NOGO > GREEN GO)**						
Middle frontal gyrus	L/R	4637	5.76	36	45	18
Occipital lobe	L/R	5484	5.60	12	−69	0
Cerebellum	L	28	3.36	−30	−63	−33
Superior frontal gyrus	L	28	3.30	−21	6	69
Middle cingulate cortex	L/R	53	3.20	−3	−24	33
Thalamus	L/R	17	2.89	−6	−9	−3
Inferior frontal gyrus/insula	R	11	2.86	36	−12	18
**INTENTIONAL ACTION > STIMULUS-DRIVEN ACTION (WHITE GO > GREEN GO)**						
Middle frontal gyrus	L/R	3445	6.25	30	24	0
Inferior frontal gyrus/insula	L	282	5.69	−27	27	0
Inferior parietal lobe	R	554	5.69	54	−48	54
Inferior parietal lobe	L	184	4.26	−54	−42	51
Precuneus	L/R	124	3.76	6	−66	42
Thalamus	L	35	3.54	−9	−15	0
Middle cingulate cortex	L/R	49	3.51	0	−24	33
Cerebellum	L	35	3.41	−33	−60	−33
**CONJUNCTION INTENTIONAL ACTION AND INHIBITION**						
Middle frontal gyrus	L/R	2625	6.15	9	24	42
Inferior parietal lobe	R	521	5.45	51	−45	45
Middle frontal gyrus	L	493	5.44	−30	51	12
Inferior frontal gyrus/insula	L	226	5.12	−30	27	0
Inferior parietal lobe	L	149	4.28	−54	−42	51
Precuneus	L/R	156	4.23	6	−66	42
Middle cingulate cortex	L/R	30	3.34	−3	−24	33
Superior temporal gyrus	R	15	2.92	54	−30	−6

**Figure 4:**
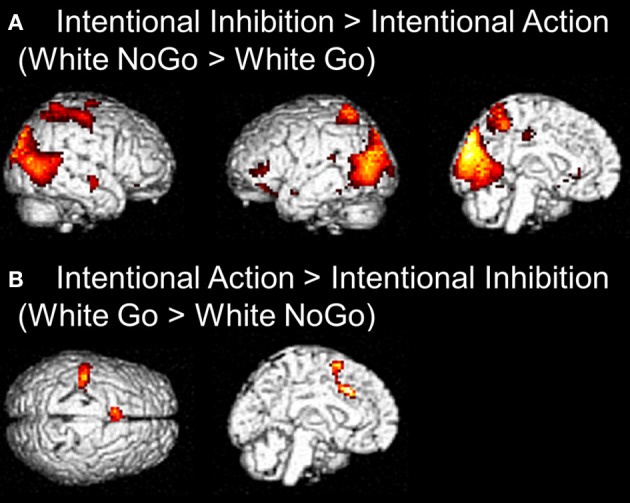
**(A)** Whole brain contrast showing activation related to intentional inhibition decision outcome (White NoGo > White Go) (FDR-corrected *p* < 0.05, at least 10 contiguous voxels). **(B)** Whole brain contrast showing activation related to intentional action decision outcome (White Go > White NoGo) (uncorrected *p* < 0.001, at least 10 contiguous voxels).

The next set of analyses focused on the intentional decision outcome. First, the brain regions underlying the intentional inhibition decision outcome were identified by computing the contrast intentional inhibition > intentional action (White NoGo > White Go). This analysis revealed activation in bilateral IPL, left IFG, left MFG, right medial temporal gyrus (MTG), and occipital lobe (see Figure 4A and Table 2). Next the reversed contrast (White Go > White NoGo) was computed to identify the brain regions underlying the intentional action decision outcome. This analysis did not result in significant activations at a FDR corrected threshold of *p* < 0.05. However, at an uncorrected threshold of *p* < 0.001 this analysis revealed activation in cingulate cortex and left postcentral gyrus, consistent with a role for left motor cortex in right-hand responding (see Figure 4B and Table 2).

**Table 2:** **Brain regions revealed by whole brain contrast, focused on decision outcomes (FDR corrected, *p* < 0.05, >10 voxels, except for White Go > White NoGo which was thresholded *p* < 0.001 uncorrected, >10 voxels)**.

**Anatomical region**	**L/R**	***K***	***Z***	**MNI coordinates**		
				***x***	***y***	***z***
**INTENTIONAL INHIBITON > INTENTIONAL ACTION (WHITE NOGO > WHITE GO)**						
Occipital lobe	L/R	5961	5.48	−15	−84	36
Superior temporal gyrus	R	51	3.88	60	−9	−9
Insula	L	34	3.59	−33	3	−12
Subgenual anterior cingulate cortex/Caudate	R	113	3.49	6	30	3
Middle cingulate cortex	L	22	3.47	−15	−24	39
Inferior frontal gyrus	L	42	3.32	−48	42	6
Superior frontal gyrus	R	33	3.27	24	−12	72
Orbital anterior prefrontal cortex	R	20	3.24	21	36	−6
Middle cingulate	R	34	3.16	15	−21	42
Inferior temporal gyrus	L	14	3.13	−42	−36	−12
Inferior frontal gyrus	L	33	3.08	−30	36	−9
Superior temporal gyrus	L	11	2.87	−60	−36	21
**INTENTIONAL ACTION > INTENTIONAL INHIBITION (WHITE GO > WHITE NOGO)**						
Anterior cingulate cortex/pre-supplementary motor area	L/R	129	4.00	−6	15	39
Postcentral gyrus	L	72	3.90	−51	−21	54

**Figure 5:**
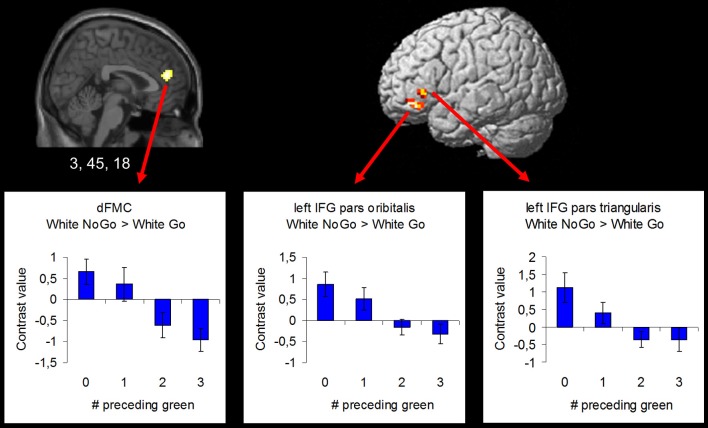
**Brain regions showing more intentional inhibition decision outcome related activation when there are less preceding green trials (uncorrected *p* < 0.001, at least 10 contiguous voxels): dFMC (3, 45, 18), left IFG pars orbitalis (−42, 39, −12), and left IFG pars triangularis (−48, 30, 0)**.

In order to examine the effect of the number of preceding green trials on intentional inhibition decision outcomes, a parametric analysis of the number of preceding green trials was performed on the contrast intentional inhibition > intentional action (White NoGo > White Go). This analysis revealed stronger activation in dFMC, left IFG pars orbitalis, left IFG pars triangularis, and right SFG when there were fewer preceding green trials (*p* < 0.001 unc.) (see Figure 5 and Table 3). ROI analysis of dFMC, left IFG pars orbitalis, and left IFG pars triangularis showed increased activation for the contrast intentional inhibition > intentional action when there were 0 or 1 preceding green trials and deactivation when there were 2 or 3 preceding green trials (see Figure 5 and Table 3). For dFMC contrast values were significantly different from zero when there were 0 or 3 preceding green trials (all *p*'s < 0.05). For left IFG pars orbitalis and pars triangularis contrast values were significantly different from zero when there were 0 preceding green trials (all *p*'s < 0.05).

**Table 3:** **Brain regions revealed by the parametric whole brain analysis on the contrast Intentional Inhibition > Intentional Action (White NoGo > White Go) (thresholded *p* < 0.001 uncorrected, >10 voxels)**.

**Anatomical region**	**L/R**	***K***	***Z***	**MNI coordinates**		
				***x***	***y***	***z***
Dorsal frontomedian cortex	L/R	79	4.28	3	45	18
Inferior frontal gyrus	L	34	3.90	−42	39	−12
Inferior frontal gyrus	L	17	3.67	−48	30	0
Superior frontal gyrus	R	10	3.51	21	33	54

#### Stop-signal task

**Figure 6:**
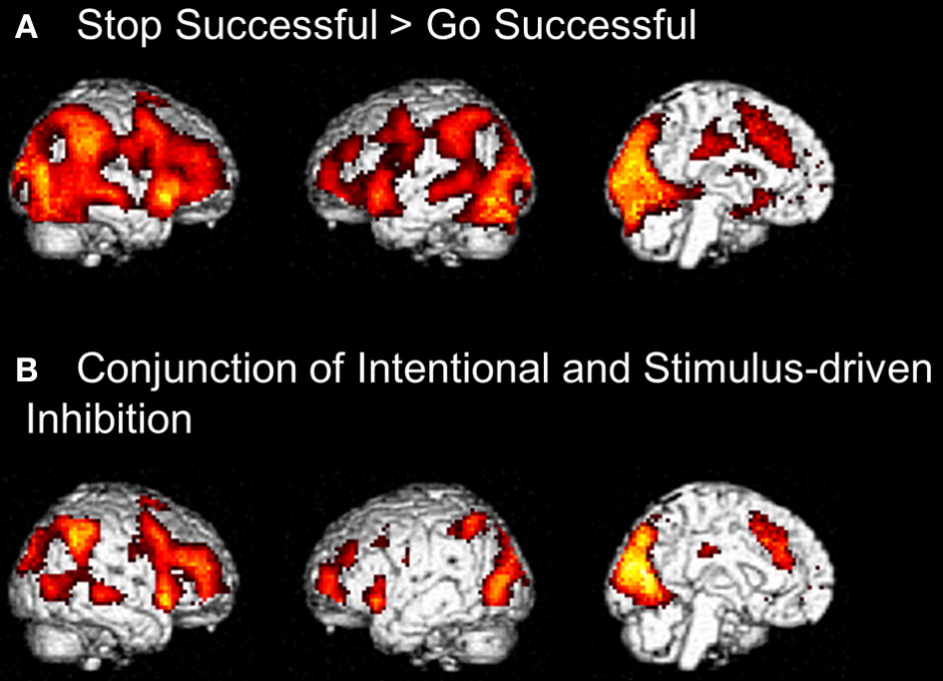
**(A)** Whole brain contrast showing activation related to stimulus-driven inhibition process (Stop Successful > Go Successful) (FDR-corrected *p* < 0.05, at least 10 contiguous voxels). **(B)** Conjunction analysis showing overlapping activation in intentional inhibition process (White NoGo > Green Go) and stimulus driven inhibition process (Stop Successful > Go Successful) (FDR-corrected *p* < 0.05, at least 10 contiguous voxels).

To identify the brain regions underlying the stimulus-driven inhibition decision process the contrast stop successful > go successful was computed. This analysis revealed activation in a widespread neural network (see Figure 6) consisting of bilateral IFG, bilateral MFG, bilateral SFG, bilateral STG, bilateral IPL, preSMA/ACC, and occipital lobe (see Figure 6A and Table 4).

**Table 4:** **Brain regions revealed by whole brain contrasts taking together intentional and stimulus-driven inhibition (all FDR corrected, *p* < 0.05, >10 voxels)**.

**Anatomical region**	**L/R**	***K***	***Z***	**MNI coordinates**		
				***x***	***y***	***z***
**STOP SUCCESSFUL > GO SUCCESSFUL**						
Lingual gyrus	L	18667	6.44	−21	−57	−6
Occipital lobe	R		6.28	27	−72	−12
Insula	R		5.72	30	18	−12
Cuneus	R		5.70	15	−96	15
Occipital lobe	L		5.67	−30	−66	−9
Insula	R		5.51	42	12	−9
Lingual gyrus	R		5.42	27	−60	−6
Calcarine gyrus	L		5.40	−6	−96	3
Inferior parietal lobe	R		5.25	48	−42	39
Middle frontal gyrus	R		5.17	36	45	21
Superior temporal gyrus	R		5.15	54	−24	−3
Caudate	R	22	3.07	12	−3	15
Caudate	L	14	2.55	−12	0	15
**CONJUNCTION INTENTIONAL AND STIMULUS−DRIVEN INHIBITION**						
Occipital lobe	L/R	4778	5.86	−45	−81	3
Middle frontal gyrus	L/R	2519	5.82	36	45	21
Middle frontal gyrus	L	311	4.66	−30	60	18
Inferior frontal gyrus/insula	L	221	4.15	−36	18	−9
Middle cingulate cortex	L/R	51	3.19	−3	−21	33
Middle frontal gyrus	L	19	2.96	−51	18	39
Postcentral gyrus	L	12	2.90	−63	−6	24

#### Comparison between intentional and stimulus-driven inhibition

Visual inspection of the intentional inhibition decision process contrast (Figure 3A) and the stimulus-driven inhibition decision process contrast (Figure 6A) suggested that there is considerable overlap in the neural networks underlying both inhibition decision processes, although the activation in the stimulus-driven inhibition contrast appears to be more extensive. In order to formally compare the neural networks underlying the intention inhibition decision process and the stimulus-driven inhibition decision process a conjunction analysis was performed. This analysis confirmed the considerable overlap in brain regions underlying both inhibition decision processes by revealing significant overlapping activation in bilateral IFG, bilateral MFG, left SFG, right STG, bilateral IPL, preSMA/ACC, and occipital lobe (see Figure 6B and Table 4).

## Discussion

The present study tested the neural correlates of intentional and stimulus-driven inhibition, using the marble task and the stop signal task. The analyses resulted in four main effects: (1) both intentional action and intentional inhibition decisions resulted in a large network of activation including the lateral prefrontal cortex, parietal cortex, and preSMA, regions previously referred to as the intentionality network (Lau et al., 2004; van Eimeren et al., 2006). (2) A parametric analysis of preceding context effects showed activation in dFMC and left IFG during intentional inhibition to decrease as the number of preceding green (Go) trials increased. (3) Conjunction analysis confirmed that the intentionality network showed large overlap with the stimulus-driven inhibition network. (4) Although the side-by-side comparison shows that intentional inhibition and stimulus-driven inhibition show overlap in networks of activation, intentional inhibition, and stimulus-driven inhibition are not directly comparable as shown by behavioral correlation analysis. Participants, who perform well on the intentional inhibition task, do not necessarily perform well on the stimulus-driven inhibition task. Thus, despite the overlap in networks of activation, behavioral performance on the intentional and stimulus-driven inhibition tasks is not predictive of each other. Below, we discuss these findings in relation to our hypotheses.

First, we aimed to replicate prior studies demonstrating that intentional action decisions are associated with increased activation in lateral and medial (ACC/preSMA) prefrontal cortex. Indeed, the contrast intentional action vs. stimulus-driven action (white vs. green marble Go responses) confirmed that this network was largely engaged, consistent with prior studies in the literature on intention action (Lau et al., 2004; van Eimeren et al., 2006). The same network was engaged in intentional inhibition decisions where we compared intention inhibition with stimulus-driven actions, further confirming the notion that this network is important for intentionality, and not for motor planning *per se* (Lau et al., 2004). This is in line with previous literature showing overlapping neural regions for inhibition and action, both when inhibition and action are internally driven (Karch et al., 2009) and when inhibition and action are externally driven (Mostofsky and Simmonds, 2008).

Secondly, we tested whether there were brain regions uniquely related to the intentional inhibition decision outcome by contrasting intentional inhibition with intentional action. A network of brain regions was active for intentional inhibition compared to intentional action including bilateral IPL and left IFG, suggesting that the inhibition process cannot be reduced to intentionality *per se* (Karch et al., 2009; Kühn and Brass, 2009). We also observed widespread activation in the occipital lobe during intentional inhibition. This is most likely due to differences between conditions with respect to the continued marble movement in the intentional inhibition vs. the intentional action trials.

Contrary to prior findings by Kühn et al. (2009), we observed no dFMC activation in this general contrast. We then explored effects of preceding context using parametric analyses. We showed that dFMC activation during intentional inhibition depended strongly on the number of preceding green trials (note that these results are based on an uncorrected threshold of 0.001, >10 contiguous voxels). At the behavioral level we also observed an effect of preceding context, such that participants were less likely to intentionally inhibit when there were more preceding green trials. Furthermore, we showed that when participants intentionally decided to act, reaction times were fastest when there were more preceding green trials. Together, these behavioral results are indicative of the formation of a disposition to act rather than inhibit, possibly reflecting an automatic associative mechanism in action generation (Perruchet et al., 2006). A run of preceding actions during green trials may progressively contribute to a predisposition to decide to act, as opposed to inhibit, on intentional white trials. This appears to reflect a positive reinforcement association for the decision to act rather than inhibit (Perruchet et al., 2006). At the neural level we showed that dFMC does show activation related to intentional inhibition, but only when following a short run of preceding instructed actions (green trials), and not following longer runs of instructed actions. Thus, veto-related activation appears to be stronger when participants are less established in a mode of prepotent responding, or set to act, to external instructive stimuli. This notion is further supported by the observation of not only increased dFMC, but also increased left IFG activation during intentional inhibition following shorter but not longer runs of preceding instructed action trials (green trials). Left IFG, like its right-hemisphere counterpart, may be critically involved in response inhibition (Leung and Cai, 2007; Swick et al., 2008).

The results of the parametric analysis shed important light on the role of dFMC in intentional inhibition, and on the significance of intentional inhibition more generally. Briefly, we found dFMC activation was reduced when previous trials had created a prepotent urge to act. Our design differs from the original free-choice whether decision of Brass and Haggard (2007), by including a large proportion of randomly-interleaved instructed action trials. These were included with the express intention of inducing a prepotent urge to act. When the prepotent urge to act is present, we reasoned that intentional inhibition should operate as a late brake on action preparation, rather than simply an early pre-decision not to initiate any action preparation at all. Interestingly, our results suggest that prepotent action also makes intentional inhibition less likely, and reduces the activation in brain areas associated with intentional inhibition. Taken together, these findings suggest that motor drive and intentional inhibition are reciprocal and antagonistic influences, analogous to the competitive interaction thought to occur between alternative response **options** (Cisek, 2007).

This reciprocal antagonism corresponds to the common intuition that inhibition of action is harder when the drive to act is strong—for example in cases such as craving and addiction. Interestingly, these are exactly the circumstances when intentional inhibition may also be most necessary. It may also explain why we did not find dFMC activation in our main contrast, while previous studies that did not use instructed action trials to enforce a prepotent urge to act did (Brass and Haggard, 2007).

Third, a **side-by-side** comparison between the intentional and the stimulus driven tasks was made. Stimulus driven inhibition resulted in the expected network of activation, including the right IFG and pre-SMA (Aron et al., 2004; Forstmann et al., 2008; Chikazoe, 2010; Aron, 2011; Jahfari et al., 2011). This network was highly comparable to the network involved in intentional inhibition, confirming that the two types of inhibition share commonalities. Both intentional and stimulus-driven inhibition require one to refrain from responding, therefore it is likely that the right IFG/pre-SMA network is important for the motoric aspect of inhibition (Chikazoe, 2010). Despite the similarities in underlying neural networks, behavioral performance on the marble and stop-signal tasks was not correlated. However, it is not uncommon that different inhibition tasks correlate poorly (Huizinga et al., 2006), but exactly how and when intentional and stimulus-driven inhibition are dissociable on the individual level remains an important avenue for future research.

Some limitations of the present study deserve mention. First, the fact that two different tasks were used to measure intentional and stimulus-driven inhibition pre-empted the possibility to compute a direct contrast between intentional and stimulus-driven inhibition. Future research might benefit from using one single task to measure both forms of inhibition, to allow for such a direct contrast. Second, for the marble task we cannot completely rule out the possibility that participants have pre-decided not to initiate an action on the intentional inhibition trials, instead of deciding in the instant to inhibit an already initiated action. The observed pattern of results showing that intentional inhibition was less likely following a run of instructed action trials, suggests that this was not the case. However, future research could shed more light on this issue, for instance by including electromyography measures to ascertain that the initial action initiation is also present in intentional inhibition trials.

Taken together, this study was the first to test the neural correlates of intentional and stimulus-driven inhibition within the same subjects. The results confirmed the hypothesis that these two types of inhibition rely on the same neural network including lateral PFC and preSMA, regions previously associated with intentionality (Lau et al., 2004; van Eimeren et al., 2006). The results also demonstrated additional activation for intentional inhibition compared to intentional action in bilateral IPL and preSMA, suggesting that the inhibition process cannot be reduced the intentionality *per se* (Karch et al., 2009; Kühn and Brass, 2009). Finally, the results showed that activation in dFMC, previously observed in other intentional inhibition studies, is dependent on specific task demands, such as prepotency of responding. Several open questions remain for how intentional inhibition relates to individual differences in self-control and self-regulation. For example, Casey et al. (2011) recently showed that individuals who can intentionally inhibit impulses to respond to immediate reward have better response inhibition associated with more lateral prefrontal cortex activation 40 years later. One of the key questions for future research is how motivational tendencies may influence internal drives to veto one's own actions when necessary.

### Conflict of interest statement

The authors declare that the research was conducted in the absence of any commercial or financial relationships that could be construed as a potential conflict of interest.
